# Unmarried women’s ways of facing single motherhood in Sri Lanka – a qualitative interview study

**DOI:** 10.1186/1472-6874-13-5

**Published:** 2013-02-06

**Authors:** Malin Jordal, Kumudu Wijewardena, Pia Olsson

**Affiliations:** 1 IMCH/International Maternal and Child Health, Department of Women’s and Children’s Health, Uppsala University Hospital, Uppsala, SE, 751 85, Sweden; 2 Department of Community Medicine, Sri Jayawardenepura University, Nugegoda, Sri Lanka

**Keywords:** Single motherhood, Unmarried mothers, Qualitative interviews, Social navigation, Sri Lanka

## Abstract

**Background:**

In Sri Lanka, motherhood within marriage is highly valued. Sex out of wedlock is socially unacceptable and can create serious public health problems such as illegal abortions, suicide and infanticide, and single motherhood as a result of premarital sex is considered shameful. The way unmarried women facing single motherhood reflect on and make use of their agency in their social environments characterised by limited social and financial support has consequences for the health and well-being of both themselves and their children. The aim of this study was to explore and describe how unmarried women facing single motherhood in Sri Lanka handle their situation.

**Methods:**

This qualitative study comprised semi-structured interviews with 28 unmarried pregnant women or single mothers. The data were analysed by qualitative content analysis and the results related to the conceptual framework of social navigation.

**Results:**

The women facing single motherhood expressed awareness of having trespassed norms of sexuality through self-blame, victimhood and obedience, and by considering or attempting suicide. They demonstrated willingness to take responsibility for becoming pregnant before marriage by giving the child up for adoption, bringing up the child themselves, claiming a father for their child, refraining from marriage in the future, permanently leave their home environment, and taking up employment. Throughout the interviews, the women expressed fear of shame, and striving for familial and societal acceptance and financial survival.

**Conclusions:**

A social environment highly condemning of unmarried motherhood hindered these women from making strategic choices on how to handle their situation. However, to achieve acceptance and survival, the women tactically navigated norms of femininity, strong family dependence, a limited work market, and different sources of support. Limited access to resources restricted the women’s sexual and reproductive health and rights, including their ability to make acceptable and healthy choices for themselves and their children.

## Background

Single motherhood is an increasing global phenomenon [1]. In many countries, single mothers risk discrimination, poverty, and lack of support [1-3]. In Sri Lanka, single mothers receive no state support, gender norms are unfavourable for single mothers [4,5], and typical women’s work is low paid [6,7] and often involves internal [8] and external migration [7,9,10]. Female education, autonomy, and marital age is relatively high in Sri Lanka [11,12] compared to other South Asian countries, and health outcomes for women and children are good [13]. However, regional and social disparities exist [14] and the ethnic conflict has had a negative impact on both women’s and men’s lives for several decades [5,15].

In Sri Lanka, women are expected to live under the protection of their families until they marry, and an unmarried women’s virginity is closely linked to the dignity of the family [6,16,17]. The Sri Lankan anthropologist Obeyesekere [17] describes how fear of shame or ‘shame-fear’ (‘lajja baya’ in Sinhala) related to sexuality and norms of proper behaviour is internalised in children from an early age. Girls are expected to conform to norms of sexual modesty, and boys are socialised into being the guardians of women’s behaviour, as they often play a public role for the family [17]. Subversion from sexual norms may result in social ridicule, reprimand and exclusion from both family and society. Thus, premarital pregnancy or merely expression of sexual knowledge before marriage can endanger both the woman’s and her family’s reputation [17,18].

Even so, premarital sex appears to be increasing [19]. Measurements of trends and distribution across socioeconomic groups in this field are difficult in absence of statistics and population based studies. However, researchers in the field points out circumstances that are likely to contribute to increased prevalence of premarital sex in Sri Lanka. Important circumstances are urbanisation, internal and external migration [8,20], increased marital age [11], and a move away from arranged marriages towards so-called ‘love marriages’ [20]. The disputed, yet internationally recognised concept of sexual and reproductive health and rights (SRHR) includes the right to reproductive decision-making and information, the means to exercise choice regarding own sexuality and reproduction, freedom from gender discrimination, and freedom from sexual violence and coercion [21]. However, in Sri Lanka, contraceptive use among unmarried couples is low due to cultural barriers and lack of knowledge on SRHR [19,22,23] and abortion is illegal unless the woman’s life is at risk [24]. Consequently, sexually active unmarried women in Sri Lanka are vulnerable to SRHR risks, including unwanted premarital pregnancies and unsafe and illegal abortions [25,26]. Sri Lanka has ratified the Convention on the Elimination of All Forms of Discrimination against Women (CEDAW) [27], declaring the intention to eliminate discrimination and violation of women’s rights [28]. However, the notion of individual’s rights often conflicts with local practices and women’s choices are affected by patriarchal power relations and hierarchical kin structures [14], and the difficulty in coping with the social demands related to sexuality and reproduction has been linked to the practice of infanticide and suicide [29-31].

Generally in Sri Lanka, motherhood is crucial to female identity, and women are perceived as natural reproducers, nurturers, mothers and wives [13,32,33]. Around one-fifth of the households in the country are headed by women [34]. The majority of these women are widowed, many of whom are war widows, divorced, or separated, and have in most cases, conceived their children within marriage [33]. Previous research has shown that widowed, divorced and abandoned single mothers are sexually, economically and socially vulnerable [35] Children normally follow the male line with regards to citizenship in a legitimate marriage, and children born outside marriage are regarded as ‘illegitimate’ [36]. Thus, while motherhood within marriage is highly valued, single motherhood as a result of premarital sex is considered shameful [5,17,36]. The stigmatised nature of unmarried motherhood renders official registration and measurement of the phenomena non-existent or unreliable. However, unmarried pregnant women in a similar setting in South India are often advised to give up their children for adoption in order to continue their lives and become “respected”, married women [37].

Facing single motherhood within this particular complex social environment, where social norms strongly condemning pre-marital sex and motherhood and virtually no social and financial security and support system other than the family, can be extremely challenging. Consequently, these women’s choices are likely to be restrained by patriarchal norms within their social environment, and the unfavourable status of children of unmarried mothers can contribute to social and health risks. How women reflect and make use of their agency, here defined as ‘the ability to set and pursue one’s own goals and interest’ [38:19] including the well-being of others and respect of social and moral norms [38], has consequences for the health and social well-being of themselves, their children, and their families.

The dialectic between individual agency and social forces are well described in the conceptual framework of ‘social navigation’ [39], where people actively ‘move within’ but are also ‘moved around’ by forces within their social environments. Agency can be actualised through either strategies or tactics, depending on the space available for manoeuvre and the agents’ personal capacity for exploiting opportunities within this space. In social navigation, strategies are understood as ‘defining and creating rules or own space’, and are often exercised by those in power, and tactics are the ‘processes of manoeuvring or bending the rules of others to one’s own advantage’ or the ‘art of the weak’ [39:133–135]. The agents’ ability to use either strategies or tactics is important for their opportunity to pursue their goals and interests and for their access to resources.

How women actualise their agency within this particular social environment can provide important clues about their ability to pursue their goals and access to resources. Thus, disentangling how women understand and tackle their situation when facing single motherhood could give useful insights into the health risks, and health promotion, for these women and their children. This study was designed with the aim of exploring and describing how unmarried women facing single motherhood in Sri Lanka handle their situation. More specifically, their perceived possibilities, difficulties, and support in their present and future life were investigated.

## Methodology

### Design and recruitment

An exploratory qualitative study design with an inductive approach with qualitative semi-structured interviews was used to enable complex descriptions from the participants’ perspective [40]. To ensure sample variation, participants were recruited at different sites: in the districts of Colombo, Gampaha, and Kaluthara, urban and semi-urban areas in the Western Province; and in Kurunegala, semi-urban and rural areas in the North Western Province. The selection criteria were being ‘unmarried woman of any age, pregnant in the third trimester or having a child less than one year of age, conceived outside marriage’. Potential participants were identified by public health midwives and nurses in district antenatal clinics and public hospitals, thus reflecting recruitment from the lower levels of the socio-economic strata. Thirty-three women were approached and informed about the purpose and procedures of the study and of the measures taken to ensure confidentiality, and that participation was voluntary. Four women declined participation, and one interview was omitted due to its short length and minimal content. Thus, the study sample consisted of 28 women.

### Participants

The age of the women ranged from 15 to 33 years, with the mean age of 23. The majority had approximately 11 years of schooling, although some had never attended school. Nine women had been unemployed when they discovered they were pregnant, and the majority of employed women had been working in factories or as housemaids when they discovered they were pregnant. Twenty women reported they had become pregnant after consensual sex, and eight after rape. Twelve (n=12) women had already given birth: 10 planned to keep their child and 2 planned to give their child up for adoption. Of the 16 women still pregnant, 9 planned to give their child up for adoption, 6 planned to keep the child, and one was undecided. An overview of the participants’ characteristics is presented in Table 1.

**Table 1:** Characteristics of participants

**Fictive name**	**Age**	**Schooling (years)**	**Occupation**	**Rape/consent**	**Delivered the baby**	**Planning adoption/ keep**
*Rangi*	15	10	Schooling	Rape	Yes	Adoption
*Kanthi*	17	10	Unemployed	Consent	Yes	Keep
*Shiromala*	18	8	Factory worker	Rape	No	Not decided
*Chamila*	18	11	Factory worker	Consent	No	Adoption
*Dayani*	19	10	Factory worker	Consent	No	Adoption
*Paba*	19	13	Unemployed	Consent	No	Adoption
*Asha*	19	11	Factory worker	Consent	No	Adoption
*Manel*	20	7	Housemaid	Rape	No	Keep
*Nisansala*	20	11	Beautician	Consent	No	Keep
*Shanti*	21	5	Unemployed	Consent	Yes	Keep
*Rupa*	21	9	Factory worker	Consent	No	Keep
*Mala*	22	2	Housemaid	Rape	Yes	Keep
*Chandrika*	22	11	Unemployed	Consent	Yes	Keep
*Neela*	23	4	Self-employed	Rape	Yes	Keep
*Suba*	23	11	Unemployed	Consent	No	Adoption
*Niroshi*	23	11	Factory worker	Consent	No	Keep
*Bama*	24	13	Unemployed	Consent	Yes	Keep
*Wathsala*	24	11	Teacher	Consent	No	Adoption
*Nalani*	24	0	Shop worker	Consent	Yes	Keep
*Inoka*	24	9	Housemaid	Consent	Yes	Keep
*Malkanthi*	24	11	Unemployed	Consent	No	Keep
*Malani*	26	8	Factory worker	Consent	Yes	Keep
*Sanduni*	28	11	Unemployed	Consent	No	Keep
*Sama*	30	0	Servant	Consent	No	Adoption
*Kala*	30	13	Unemployed	Consent	Yes	Adoption
*Reka*	31	11	Factory worker	Rape	Yes	Keep
*Kema*	31	11	Unemployed	Rape	No	Adoption
*Reeta*	33	11	Housemaid	Rape	No	Adoption

### Data collection and handling

Sri Lankan medical graduates and social scientist students, fluent in both spoken and written English and trained in qualitative data collection, organised the recruitment and conducted the interviews in Sinhala or Tamil. The topics covered in the interviews included life situation before pregnancy, realising the pregnancy, difficulties and possibilities in present and future life, and support from partner, family, and society. The interviews had a conversational style to enable the interviewee’s to talk and reflect. All interviews were audio-recorded and transcribed into text. The same research assistant who conducted the interview translated the text from Sinhala or Tamil into English. Another research assistant checked the audio recording against the text. The interviews lasted between 12 and 75 minutes and provided 16 hours of audio-recordings.

### Data analysis

To obtain the complex and rich descriptions of the participants diverse views, qualitative content analysis [41] was chosen as the analytical method. Qualitative content analysis aims at grasping the manifest and latent messages in the texts, a process that involves multiple readings of the text and identifying meaning units or sequences of importance for the study aim. The meaning units were condensed and further shortened into codes, which were finally grouped into categories and sub-categories based on similarities in the manifest content. An illustration of the process of analysis is presented in Table 2.

**Table 2:** Illustration of the analysis process

**Meaning unit**	**Condensed meaning unit**	**Code**
Interviewer (I): What did you feel when you got to know that you were pregnant?	The despair when realizing the pregnancy made her consider suicide, but when consulting a friend she was encouraged to inform the family and discard suicide	Considered and discarded suicide*
Participant (P): I felt like ‘Oh! What’s to be done’? I felt like committing suicide.		
When I told that to my friend, she asked me not to do such a foolish thing, but to inform my family, and said that they wouldn’t let me down. So I thought		
I shouldn’t do that [commit suicide] and informed my family instead		
I: Although you thought of committing suicide you didn’t attempt it, did you?		
P: No		

* The code was later part of the category ‘Expressing awareness of having trespassed norms of sexuality‘.

### Ethical considerations

The interviews were held in privacy to ensure confidentiality. Before giving their informed oral consent, participants were provided oral and written information about the procedure of data collection, confidentiality, and voluntary participation, including their right to withdraw from the study at any time. Permission to recruit participants was obtained from the heads of the hospitals and the Medical Officers of Health in each district. Ethical clearance (Number A 244) was obtained from the Ethical Review Committee, Faculty of Medical Sciences, University of Sri Jayewardenepura, Nugegoda, Sri Lanka. The Regional Ethical Review Board in Uppsala, Sweden, conducted a consultative ethics review. The women are presented with pseudonyms in order to safeguard their confidentiality.

## Results

The women became pregnant before marriage and within a social environment characterised by strong family dependency, poverty, limited employment opportunities, the idea of a mother as self-sacrificing, and social norms condemning premarital sex. Their relationships with the man who fathered their children were complicated, in most cases over and seldom supportive. The women had not used contraceptives or attempted to prevent the pregnancy in other ways, and reported this to be due to being unable to foresee the sexual act, being raped, having limited sexual knowledge, assuming the partner would take responsibility, hoping the pregnancy would lead to marriage, or not considering the possibility of becoming pregnant. Some women had tried to abort the pregnancy through traditional methods, but without success. The reasons presented by the women who had not tried to terminate the pregnancy were delay in realising the pregnancy, perceiving abortion a sin, wanting to become a mother, seeing the child as a product of love, expecting to marry their ex-partner, or hoping the birth of the child would bring the partner back. The ways the women handled their situations when facing single motherhood are presented in two categories and nine sub-categories in Table 3.

**Table 3:** Results presented in categories and sub-categories

**Categories**	**1 Expressing awareness of having trespassed norms of sexuality**	**2 Taking responsibility for having become pregnant before marriage**
Sub-categories	1.1 Hide the pregnancy	2.1 Give the child up for adoption
	1.2 Demonstrate self-blame, victimhood and obedience	2.2 Bring up the child 2.3 Claim a father for the child
	1.3 Consider and attempt suicide	2.4 Refrain from marriage
		2.5 Permanently leave one’s home environment
		2.6 Seek employment

### Expressing awareness of having trespassed norms of sexuality

The fear of shame due to having trespassed social norms of sexuality on becoming pregnant before marriage dominated the interviews. In order to avoid exclusion from family and society and to ensure their financial and social survival, the women attempted to seclude their pregnant conditions for their social environments. When unable to do so, the women tried to reduce the shame and minimise the reactions of families and society by expressing awareness of having trespassed important social norms through demonstrating self-blame, victimhood and obedience, and by considering or attempting suicide.

#### Hide the pregnancy

A publicly known premarital pregnancy was strongly believed to negatively affect the woman’s and her family’s dignity. The women tried to hide their pregnant conditions from neighbours, employers, ex-partners, and relatives, including their own parents, due to fear of social ridicule, reprimand and exclusion from their families. However, the difficulty in concealing the pregnancy from their closest family members and the need for support meant the majority informed at least one person, often their own mother. With or without assistance, the women sought shelter inside their own homes, with distant relatives, or with faith-based organisations (FBO) recognised as ‘safe havens’ for unmarried pregnant women:

Participant: Because I am here [FBO], I don’t have much of a problem. There is a situation that I can’t face the society if I go home.

Interviewer: Now do the neighbours in the village know this?

Participant: Nobody knows, only my family knows this. (Wathsala, 24 years)

#### Demonstrate self-blame, victimhood, and obedience

When informing others about their pregnancy, the women encountered different reactions; some were supported and emotionally soothed, whereas, others were scolded, threatened and punished. In the hope of reducing the risk of rejection and condemnation, the women demonstrated self-blame, regret and guilt when facing their family members, employers, friends, or FBO or health staff, irrespective of having consented to sex or not. The women blamed themselves for having agreed to sex or exposing themselves to rape, and demonstrated remorse when scolded by family members:

They [the family] scolded me very severely. I didn’t say a single word. I kept my mouth shut. Because I did the wrong thing. (Kala, 30 years)

The women portrayed themselves as victims of dishonest and calculating men as part of demonstrating awareness of having trespassed sexual norms. In doing so, the women acted in accordance with a social environment where women are depicted as submissive, trustful and helpless in relation to men and sexually active unmarried women are regarded as both promiscuous and immoral. The women described themselves as morally upright women who had been abandoned by a dishonest man after being persuaded to have sex, as association with promiscuity meant risk of being excluded from kin and society:

He worked in a bus. I went to Colombo on that bus. That’s how I met him. Then we had an affair. I asked him to marry me. Then he took me somewhere far away and we married. Signed the documents. After that, I came home. That very day his relatives came to my house and told my mother that he was a married man and to ask me to stop the affair. They had found one of my letters. She told me that we didn’t want to shatter a family and ask me to stop. Then he told me that he was a father of three. Isn’t that a little late to tell the truth? (Sanduni, 28 years)

The women described themselves as financially, socially and/or emotionally dependent on their families, employers or relatives, and demonstrated obedience in an attempt to reduce the risk of rejection. Thus, obedience was displayed as a way of complying with family members’ demands and rehabilitating their relationships as daughters.

Interviewer: Will you bring up the child?

Participant: I haven’t decided about it; I have to stay with my mother, so I have to obey with what ever my mother says. (Shiromala, 18 years)

#### Consider and attempt suicide

Some women considered and attempted suicide out of fear of shame related to having become pregnant prior to marriage. Suicide was perceived as a means of escape from their shameful conditions, and communicating suicide was a way of expressing awareness of the gravity of the situation and attaining support. While the majority of women who had considered suicide re-evaluated the situation and discarded suicide as a solution, one woman had attempted suicide by drinking pesticides:

Interviewer: Did you intend to attempt suicide or what’s the reason for an act like this?

Participant: Yes, I attempted suicide by drinking that [pesticide].

Interviewer: What is the reason for attempting suicide?

Participant: You know I realised only after developing this pain that I had become pregnant. (Mala, 22 years)

### Taking responsibility for having become pregnant prior to marriage

How women reflected on what to do after the baby was born depended on the reactions they received within their social environments, their personal capacity, and on the time allowed for reflection. The women all demonstrated willingness to take responsibility for becoming pregnant before marriage, although in different ways. This involved planning to have the child adopted, bring up the child themselves, claim a father for their child, refrain from marriage in the future, permanently leave one’s home environment, or take up employment. In doing so, the women hoped to be accepted by their family and society, and ensure their financial and social survival.

#### Give the child up for adoption

One way of taking responsibility for having become pregnant prior to marriage was to have the child adopted. Adoption was often encouraged by family members, employers, friends, and health and FBO staff and a degree of coercion was involved:

Participant: As I am very young she [participants’ mother] asked me to give the baby for adoption (…) She says it is difficult to raise a child without a father and I will have to face lot of problems, so it’s better to give it for adoption.

Interviewer: Would you like to give the child for adoption?

Participant: Really, I don’t like it. But everybody asked me not to keep him with me and they threaten to kill me. They do not want me to come to the village with the child… Anyway, I cannot stay here alone. (Dayani, 19 years)

If the pregnancy was not publicly known, having the child adopted enabled the woman to return to her community with her reputation intact; thus, the woman remain marriageable, and was something that was regarded as the best option for both the woman and her family. Moreover, having the child adopted enabled the child to grow up in a two-parent family instead of growing up with a stigmatised, unmarried mother. Most women expressed reluctance towards giving up their child, but were motivated to safeguard their own and their family’s dignity, as they were advised, pressured or threatened with murder and family exclusion. Without support to keep their child in their social environment, adoption was considered the only realistic option:

It is difficult to take care of my child. Most people advise me to give the child up for adoption. Madam [employer] also told me so, and she told me that she will help with the adoption. She is very angry with me because I didn’t tell her about my sexual relationship with that boy. She is helping me… she came here and gave me some clothes, and money too. I believe that she will help me during the delivery also. I have only her help. Other than that, no one helps me. (Sama, 30 years)

#### Bring up the child

Although having the child adopted was one way of taking responsibility for having become pregnant, bringing up the child by themselves was another option for these women. For some women, bringing up the child was considered a mother’s responsibility, and even if this provoked social shame, some women perceived this option as being in the best interest of the child. This was in accordance with gender stereotypes of women as self-sacrificing, loving and caring mothers, although it was not compatible with the norms of female chastity and sexual innocence. For some women, support from their families for keeping the child was the most important factor determining the women’s decision; this gave them strength to withstand social ridicule and provided physical and financial security. Others, who had not initially received support, had actively identified other forms of support within their social environment, including friends, neighbours, health personnel, and FBO; thus, the women planned to keep the child without support from their families. Although anticipating shame and difficulty as unmarried mothers, some women argued the emotional attachment between mother and child made adoption impossible:

Though the convent says that they are going to give the baby… (…) although I told so at that time, when I see the other children, I don’t feel like giving up my [own] baby. I love the baby a lot. It is really hard to become a mother. I don’t think I will be able to give up the baby. (Nisansala, 20 years)

#### Claim a father for the child

In a social environment where a child ‘without a father’ is considered illegitimate, some women adopted different ways of ‘providing’ a father for their child. This involved leaving the child with its biological father and his wife, or keeping the child but bring the responsible man to court in hope of making him accept paternity. Although going to court could force the man to support the woman and the child financially, this was perceived of minor importance in relation to obtaining a father’s name on the child’s birth certificate, as this demonstrated he accepted fatherhood:

Participant: I will raise the baby if he accepts that the baby is his. If he doesn’t I will definitely sue him. Really, I don’t want anything from him. I can raise the child on my own. I don’t want even 5 cents from him. But I can’t bring a child who doesn’t have a father into the world (becomes tearful). So somehow or the other I will sue him.

Interviewer: What do you claim for when suing him?

Participant: Only for him to sign the birth certificate (…) I don’t want any compensation in form of money. All I want is for him to accept paternity. I can’t give birth to a child who does not have a father. (Niroshi, 23 years)

#### Refrain from marriage

Many women planning to have their child adopted expressed reluctance towards getting married in the future due to their negative experiences with men. Thus, the women conveyed willingness to sacrifice their possibility of becoming ‘respected married women’ in order to take responsibility for having engaged in premarital sex. Although not all women who planned to have their child adopted were opposed to the idea of getting married in the future, reluctance towards hiding their past, fear rumours about the pregnancy would leak out, concern about re-traumatisation if they had another child, and reluctance to trust a man again, were the main arguments for remaining single:

Participant: The sisters [at FBO] told me that they would arrange a marriage if they find a good person. But I don’t feel like marrying.

Interviewer: Why is that?

Participant: After being cheated once, it is difficult to trust men. I feel that everybody is the same. (Chamila, 18 years)

Some women keeping their child hoped to marry, mostly for their own and their child’s protection; however, they anticipated there was little chance of finding a man willing to marry an unmarried mother. Other women expressed reluctance towards marrying, due to their negative experiences with men and out of concern for their child’s well-being. The biological connection between father and child was perceived important for a man’s ability to love a child fully; consequently, some women considered marrying a man other than the child’s biological father could hamper the child’s future well-being:

No, I will not marry anybody. I don’t like to. If I get married what will happen to this child? They will say that they love him, but when I’ll get another baby, one usually do when marrying again, they will accuse this child of being a ‘bastard’ and ill-treat him.(Neela, 23 years)

#### Permanently leave one’s home environment

Some women planned to leave their homes permanently, having jeopardised their own and their family’s dignity by becoming pregnant before marriage. Migration enabled them to escape the social shame attached to the premarital pregnancy and conceal the shameful aspects of their past, and if they planned to keep their child, pretend to have conceived within marriage. The women also considered their family members’ dignity and siblings’ future marriageability when planning to migrate:

When looking at my family members [siblings], they are all at the age of studying. I have to think of their future as well. If I am to do so, I will have to live far away from them and bring up the child. (Nisansala, 20 years)

#### Seek employment

The women’s social environment was characterised by poverty and limited work opportunities for women, and compulsory migration in typical women’s jobs. On becoming pregnant, the women had jeopardised their families’ economic well-being through being an extra burden and mouth to feed, or if they had been the main breadwinners, halting their family’s income. The women planned to take up paid work after the delivery, and take responsibility for their own, their child’s, or other family members’ well-being and survival. Home-based employment, such as vegetable cultivation or pottery production enabled them to remain in the village, although with a marginal income. Migration to one of the Free Trade Zones (FTZ) in Sri Lanka or to the Middle East would expand earning possibilities, but involved leaving their familiar surroundings and leaving their child behind. Home-based employment and internal migration to FTZ was perceived as compatible with bringing up their child:

I have already found a day care to keep him [the baby], to keep him at the daytime only, so that I will be able to work in the garment factory [in the FTZ] during the daytime. He will be with me. That is the plan for the future. He will not be given for adoption. (Neela, 23 years)

Although the majority of women expressed willingness to take up paid work, some planned to rely financially on family members or an FBO. One woman planned to dedicate her life to orphans and work in an orphanage as a way of compensating for not being able to bring up her child herself:

After I hand over my baby, I am hoping to leave this place and go to an orphanage and work with orphans. That’s what I am hoping to do in the future. (Suba, 23 years)

## Discussion

The interviews in this study aimed to explore how unmarried women facing single motherhood in Sri Lanka handled their situation. The women were expressing awareness of having trespassed norms of sexuality and in different ways planned to take responsibility for having become pregnant before marriage. Throughout the interviews, the women expressed fear of shame, and a striving for both familial and societal acceptance and social and financial survival. The results will be discussed in the light of social navigation and the implications for the women’s SRHR.

### Social navigation when facing single motherhood

The conceptual framework of social navigation [39] can be used to gain further understanding of the information gained from the interviews and how these women actualised their agency in relation to their social environment, and how this affected their ability to pursue their goals. Through anchoring the women’s agency and actions in the theoretical distinction between strategies and tactics within social navigation [39], tactics being the process of manoeuvring or bending the rules of others to one’s own advantage, and strategies being defined as creating rules or own space [39], several tactics, but no strategies, were identified. The women in the present study had limited possibilities for shaping social processes, but rather tried to ‘grab on to possibilities arising in the shadow of the strategic others’ [39:134]. The reasons for considering these actions as tactics, and not strategies, are explained below with a suggestion for what this means for their access to resources and ability to pursue their goals.

Fear of shame was a major concern related to having trespassed social norms of sexuality, and was reflected in the way the women took responsibility for having become pregnant before marriage: this confirmed Obeyesekere’s [17] description of ‘shame-fear’ as an important part of the socialisation of women in relation to sexual norms in the Sinhala culture. The fear of shame greatly influenced the women’s feelings and actions and claiming victimhood was one way of reducing the shameful aspect related to their premarital sexual history. Through portraying themselves as victims who had been persuaded and agreed to have sex out of love, trust and the promise of marriage, the women distanced themselves from women who have sex out of promiscuity. The claiming of victimhood was in accordance with gender stereotypes in Sri Lanka [42] that portray women as subject to betrayal by men: this adherence to gender stereotypes of proper femininity was in agreement with the desire to ‘pass as normal’ when carrying signs of stigma [43]. In the context of Sri Lanka, this can be connected to the notion of ‘respectability’ that is particularly important [4], and for unmarried women to be associated with chastity and virginity [6,18]. The women in the present study portrayed themselves as respectable, unmarried women who had been tricked into sex, thus, hoping to ‘pass as normal’ and enhance acceptance by their families and society. In India, unmarried women also claim victimhood to repair their damaged reputation [37]; however, Bos [37] considers this ‘a strategic way of creating space and a degree of self-respect within a rigid framework of social structures and ridicule for unmarried mothers’ [37:193]. Although we agree unmarried pregnant women claim victimhood to gain self-respect within rigid social structures, we suggest that women, who take on an obedient and fearful attitude in order to restore a damaged reputation, do not create their own space. Thus, this is not a strategic move, but the use of a tactical agency or ‘bending the norms of others’ (in this context, gendered norms and patriarchal power structures) to their own advantage.

The fear of shame was present in accepting the consequences of becoming pregnant before marriage, for example when planning to have the child adopted or migrate permanently, with or without their child. Adoption and migration was considered through concern for their families’ shame and dignity. The family’ reputation is an important reason for unmarried women in Sri Lanka to seek illegal abortions [25]. However, while the option of having the child adopted due to family pressure or respect for social norms, or permanently leave their homes and natal families to safeguard their family’s reputation reveals agency, it does not demonstrate a strategy as defined as the ability to create own space and define own rules. Instead, the women’s limited capacity to pursue their own personal interests should be considered as being moved around by external forces, thus, the option of giving the child up for adoption was a tactical manoeuvre within their limited social space.

The women’s social environment were characterised by both strong social norms condemning premarital sexual relations and the ideal of a mother as self-sacrificing, loving, and caring nurturer. Accordingly, the women planned to keep and bring up their own child as a way of taking responsibility for their premarital pregnancy. Through emphasising emotional attachment and biological connection with their child, the women argued that pursuing their responsibility as mothers was more important than their potential suffering of shame and an insecure future with their child. Thus, in accordance with the existing ideals of motherhood and gender stereotypes portraying mothers as natural nurturers of their child [5,32,33], some women weighed this against the negative aspects of becoming pregnant before marriage. Similarly, unmarried mothers in India appear to weight their choices of whether to relinquish or keep their child between two different types of shame; the shame related to the premarital pregnancy and the shame of giving away one’s child [37]. Although the women perceive their status as ‘unmarried and pregnant’ shameful, the situation appears to change after childbirth, where the women came to see themselves as ‘being a mother of a child’ and unmarried pregnant women who initially planned to have their child adopted changed their mind after the child was born [37]. Similarly, in the present study, the women revealed they changed their mind as the pregnancy proceeded, and their growing feeling of being ‘mothers’ rendered the option of adoption unbearable. However, although the women’s ability to keep their child suggested capacity for making choices, the gendered norms and structural barriers in the women’s social environment left little space for them to live safe and satisfying lives together with their child.

The women indicated a variety of plans to seek employment for financial survival and to take responsibility for themselves and their families. This involved migrating to the FTZ, with or without their child, or work as housemaids in the Middle East, something that involved having the child adopted or leaving it behind with their families. Although these actions could be considered strategic, we argue the constraints they face on the labour market, including the pressure to migrate [9], limited earning possibilities, and potential social stigma as working and unmarried mothers [1,3] renders this as a tactical manoeuvre within their social space.

Through the conceptual framework of social navigation, the way women use agency despite living under highly gendered and patriarchal circumstances could be illustrated. The perception of women’s actions as tactics, rather than strategies, facilitates understanding of and acknowledgement of their agency, while recognising both their limited access to resources and their disadvantaged position in society. Although the women actively explored their social environment for possibilities, their possibilities were largely dependent on the choices of powerful others. The ‘powerful others’ being hierarchical families, patriarchal gender structures, powerful employers and limited work opportunities, restricted the women’s choices and limited their ability to pursue their own personal interests. Only when the women received support for keeping their child or had personal capacity to leave their home environment did they feel able to keep their child, something that was often articulated as a wish, but seldom as a right. Instead, the women felt obliged to conform to ideals of social norms, the well-being of their families, and to patriarchal structures within their society to avoid social and familial exclusion and to ensure their and their children’s survival.

### Sexual and reproductive health and rights implications

These women’s choices were restricted by structures within their social environments, and these restrictions had implications for their SRHR.

Several women in this study were raped, a serious SRHR violation. In Sri Lanka, rape is reported as an increasing problem, especially in relation to migration [9]. SRHR can be jeopardised even for women who conceive in mutual relationships, for example by entering a fake marriage or being persuaded to have sex by their partners’ promise of marriage. A lack of reproductive health education for young people [44] and rigid gender norms prescribing unmarried women to be virgins [8,14,45] contribute to reduced capability for women to access reproductive health information. Although there are no legal restrictions within the health services hindering unmarried women to obtain contraceptives, the stigmatisation of unmarried women who demand, obtain or use contraceptives [8] has negative consequences for their right to health promoting choices. Once pregnant, the women face legal, religious, cultural and knowledge barriers to abortion, and difficulties in coping with social and cultural demands, including accusations of premarital sexual relations, are associated with a high female suicide rate in Sri Lanka [29,30]. The tendency for women in the present study to consider or attempt suicide as the solution to their situation should be considered an indicator of the violation of their SRHR. Further, the women were torn between raising their child with concerns for their own and their child’s future, or surrender their child due to pressure from kin, lack of financial means and prevailing gender structures, but with emotional suffering. The lack of a state support system for protecting unmarried mothers and their children renders these women dependent on family support and leaves them vulnerable to discrimination and marginalisation in society.

Essential requirements to improve women’s SRHR in Sri Lanka should include access to reproductive health education, information, and services, availability of contraceptives and provision of safe and affordable abortion supported by informed counselling. This approach should be gender sensitive, in other words also available and suitable for men as well, increasing their knowledge as a means to hold them equally responsible for sexual activity and its consequences. Improved employment opportunities would increase the women’s position in society, and economic security through governmental and/or non-governmental financial assistance could empower single mothers to make important decisions regarding themselves and their children. Furthermore, it is important to create awareness of how gendered norms work as barriers to unmarried women’s SRHR and child health and rights.

### Methodological considerations

The qualitative study design was chosen as this area is under-researched and required in-depth exploration. This approach was feasible because the participants belong to a ‘hidden’ group of women, which makes the selection of a larger sample difficult. The design also facilitated the exploration of the nuances of women’s agency and the constraints they faced within their social environments.

Conducting cross-cultural qualitative research implies several challenges and limitations [46]. Limitations could include little variation within the sample, use of several data collectors, and interpretation of data analysed by researchers with different cultural backgrounds. Throughout the process, measures were taken to address these limitations and to ensure quality and trustworthiness [42].

The group of participants included women from different age groups and geographic areas. This helped to capture the complexity of the phenomenon and gave rich data that conveyed understanding of the research question from different perspectives. The inclusion of both women who had lived with their child for up to six months and those who were still pregnant provided a complex and nuanced picture of the phenomenon.

The composition of the research team, including both insiders and outsiders to the Sri Lankan context created opportunities challenges throughout the research process. The challenges included limited understanding of Sri Lankan cultures and languages from the Swedish part of the research team. This gave the team many opportunities to discuss multiple perspectives, which increased the cultural understanding and achievement of consensus on the meaning of the findings. To ensure consistency in the questions and analysis, the same main investigators were maintained throughout the study process. Furthermore, the same overall topic guide was used in all interviews and efforts were made to supervise and support the research assistants. To help the reader judge the researchers’ interpretations and the transferability of the results to other settings we have provided detailed and referenced descriptions of the context and culture, characteristics of the participants, and a rich and vigorous presentation of the phenomena with illustrative quotations.

As the participants were only interviewed once, we were not able to explore whether and how their perceptions changed with time. A follow-up study with the same participants would provide deeper understanding on whether and how their positions and choices change, and how they experience their lives after making their decision. We analysed these single mothers as a unitary group, irrespective of pregnancies following consensual sex or rape. However, it would be valuable in future studies to address the distinctive challenges and experiences of these two groups. Differences between women planning to keep their child and those planning relinquishment could also be explored in another study with a larger study sample to provide further information on the differences in women’s resources that could be used in intervention programs.

## Conclusion

A social environment highly condemning of unmarried motherhood hindered these women from making strategic choices on how to handle their situation. However, to achieve acceptance and survival, the women tactically navigated the norms of femininity, strong family dependence, a limited work market, and different sources of support. Limited access to resources restricted the women’s SRHR, including their ability to make acceptable and healthy choices regarding themselves and their children.

## Abbreviations

FTZ: Free Trade Zone; SRHR: Sexual and Reproductive Health and Rights; FBO: Faith Based Organisation; ICPD: International Conference on Population and Development; CEDA: Convention on the Elimination of All Forms of Discrimination against Women; SIDA: Swedish International Development Cooperation Agency.

## Competing interests

The authors declare that they have no competing interests.

## Authors’ contribution

MJ was engaged in data collection, data analysis, and the drafting of the manuscript. PO and KW designed the study, organised data collection, and took part in the process of analysis and drafting of the manuscript. All authors, MJ, PO and KW, had access to the data and are responsible for its integrity, and read and approved the final manuscript.

## Pre-publication history

The pre-publication history for this paper can be accessed here:

http://www.biomedcentral.com/1472-6874/13/5/prepub
